# Comparison of Incidence of Myocardial Infarction in Patients With Rheumatoid Arthritis and Diabetes Mellitus

**DOI:** 10.7759/cureus.15716

**Published:** 2021-06-17

**Authors:** Azka Ali, Akhtar Ali, Dilpat Kumar, Ravi Kumar, Kanwal Elahi, FNU Suman, Zauraiz Anjum, Payal Tharwani, Maha Jahangir, Amber Rizwan

**Affiliations:** 1 Internal Medicine, Mayo Hospital, Lahore, PAK; 2 Pulmonology and Critical Care, Services Hospital, Lahore, PAK; 3 Internal Medicine, Western Michigan University, Kalamazoo, USA; 4 Internal Medicine, Chandka Medical College, Larkana, PAK; 5 Internal Medicine, Jinnah Postgraduate Medical Centre, Karachi, PAK; 6 Internal Medicine, Peoples University of Medical and Health Sciences for Women, Nawabshah, PAK; 7 Internal Medicine, Fatima Jinnah Medical University, Lahore, PAK; 8 Oral Medicine, Isra University, Hyderabad, PAK; 9 Internal Medicine, Dow, Medical College, Dow University of Health Sciences, Karachi, PAK; 10 Family Medicine, Jinnah Postgraduate Medical Centre, Karachi, PAK

**Keywords:** rheumatoid arthriitis, diabetes, major adverse cardiovascular event, association, risk factor

## Abstract

Introduction

Patients with rheumatoid arthritis (RA) have a higher risk of cardiovascular diseases (CVDs) when compared to the general population, with most deaths attributed to myocardial infarctions (MI). However, patients with RA do not get the same attention in terms of cardiovascular screening as compared to other diseases, like diabetes mellitus (DM). Therefore, this study aims to compare the risk of CVD among patients with RA and DM.

Methods

This prospective study was carried out in Pakistan’s two tertiary care hospitals. A total of 750 participants were enrolled in three groups with a 1:1:1 ratio; patients with RA, type 2 DM, and the control group. Patients were observed for 12 months or until the development of a major adverse cardiovascular event (MACE), whichever occurred first.

Results

Both fatal (12.66% vs. 13.48%; p-value: 0.79) and non-fatal (3.93% vs. 4.35%; p-value: 0.82) MI was comparable between both RA and DM group. However, compared to the control group, non-fatal MI (12.66% vs. 5.58%; p-value: 0.01) was significantly higher in the RA group.

Conclusion

Our study shows that RA and DM have an equal risk of cardiovascular (CV) events. It is important that RA should be considered as a prominent risk factor for CV events. The management of these patients should be multidisciplinary, including cardiologists.

## Introduction

Rheumatoid arthritis (RA) is an autoimmune chronic inflammatory disease of the synovial membrane of the joints. It affects approximately 0.5% to 1% of the population globally, mostly the elderly, with a predilection towards the female sex. Chronic inflammation leads to joint pain, stiffness, and joint distortion, leading to restricted movement and progressive disability that greatly hampers the quality of life in these individuals [1,2]. RA also presents with extra-articular manifestations like osteoporosis, pericarditis, vasculitis, and Sjögren’s syndrome [3].

Patients with RA also have a higher risk of cardiovascular diseases (CVDs) when compared to the general population, with most deaths attributed to myocardial infarctions (MI) [4]. This increased risk could be due to the concomitant presence of other risk factors like hyperlipidemia, hypertension, diabetes mellitus (DM), poor physical fitness, or simply from the chronic inflammatory state of the body [5,6]. DM is a well-established risk factor for CVD, particularly coronary artery disease. It increases the risk of CVD two to three folds compared to the non-diabetic population [7,8]. The risk of CVD in RA has been compared to that of DM and the scarcity of literature suggests consistent results so far [9-11]. A study reported that patients with DM have higher cardiovascular (CV) mortality rates when compared to RA, however, the difference was not statistically significant in >75-year-old patients, while another study found the comparable prevalence of CVD between the two disease groups [12,13]. Both RA and DM certainly contribute to the increased CVD risk, but more studies are needed to ascertain the extent to which the risk of CVD differs between the two. The level of awareness regarding RA as a risk factor is low when compared to DM [12].

This discrepancy could lead to physicians not recommending the uptake of preventive measures for CVD in RA patients as actively done in patients with DM. Therefore, the awareness of the comparative risk of CVD in RA and DM is essential to guide management accordingly and reduce the morbidity and mortality in these groups. Therefore, this study aims to compare the risk of CVD among patients with RA and DM.

## Materials and methods

This prospective study was carried out in Pakistan’s two tertiary care hospitals from January 2019 to March 2021. From the rheumatology outpatient department, 250 patients with a confirmed diagnosis of RA, with no history of DM, were included in this study. From the outpatient department of the cardiology unit, 250 participants with type 2 DM, in the absence of a known diagnosis of RA, were incorporated in the diabetic group. The control group enrolled 250 participants without any history of type 2 DM and RA. Patients were enrolled via non-consecutive convenient probability sampling. Informed consent was taken from participants and ethical review board approval was taken before the start of enrollment.

Risk factors including age, BMI, hypertension, smoking, past and family history of MI were noted using a self-structured questionnaire. Patients were observed for 12 months or till the development of MI. MI was diagnosed based on symptoms, cardiac enzyme, and ECG. Patients lost to follow-up from the RA, diabetic, and control group were 21, 20, and 17, respectively.

Statistical analysis was done using the Statistical Package for the Social Sciences (SPSS) v. 23.0 (IBM Corporation, Armonk, New York, USA). Continuous variables were analyzed via descriptive statistics and were presented as mean and SD. Categorical data were presented as frequency and percentages. To compare demographics for all three groups, ANOVA was applied, whereas chi-square was used to compare events between various groups as appropriate. With the help of an online calculator (MedCalc.org), the RR was calculated to compare the incidence of MI between the diabetic and RA groups, and between RA and control groups. A p-value of less than 0.05 meant that the difference between the groups is significant and the null hypothesis is void.

## Results

The characteristics and risk factor profile were similar between both groups (Table 1).

**Table 1:** Demographics and risk factor profile of participants with RA, DM, and control group. DM: Diabetes mellitus; MI: Myocardial infarction; NS: Nonsignificant; RA: Rheumatoid arthritis.

Characteristics	Participants with RA (n = 229)	Participants with DM (n = 230)	Control group (n = 233)	P-value
Age in years (Mean ± SD)	45 ± 11	47 ± 12	47 ± 11	NS
Male (%)	112 (48.91%)	120 (52.17%)	119 (51.07%)	NS
BMI greater than 30 kg/m^2^ (%)	51 (22.27%)	48 (20.87%)	54 (231.8%)	NS
Cholesterol level greater than 200 mg/dL (%)	112 (48.91%)	110 (47.83%)	109 (46.78%)	NS
Hypertensive (%)	151 (65.94%)	154 (66.96%)	152 (65.24%)	NS
Current smokers (%)	92 (40.17%)	89 (38.70%)	90 (38.63%)	NS
Previous history of MI (%)	10 (4.37%)	08 (3.48%)	08 (3.43%)	NS
Family history of MI (%)	12 (5.24%)	11 (4.78%)	11 (4.72%)	NS

Fatal (12.66% vs. 13.48%; p-value: 0.79) and non-fatal (3.93% vs. 4.35%; p-value: 0.82) MI was comparable between both RA and DM groups. However, compared to the control group, non-fatal MI (12.66% vs. 5.58%; p-value: 0.01) was significantly higher in the RA group. The RR for non-fatal MI in the RA group was 2.226 compared to the control group, whereas the RR for fatal MI in the RA group is 3.05 compared to the control group (Table 2).

**Table 2:** Comparison of fatal and non-fatal MI between the RA, DM, and control group. DM: Diabetes mellitus; MI: Myocardial infarction; RA: Rheumatoid arthritis. A: Chi-square applied between the RA and DM group. B: Chi-square applied between the RA and control group.

Characteristics	RA group (n = 229)	DM group (n = 230)	Control group (n = 233)	RR^A^ (CI: 95%)	P-value ^A^	RR^B^ (CI: 95%)	P-value ^B^
Non-fatal MI (%)	29 (12.66%)	31 (13.48%)	13 (5.58%)	0.93 (0.58-1.58)	0.79	2.26 (1.21-4.25)	0.01
Fatal MI (%)	9 (3.93%)	10 (4.35%)	3 (1.29%)	0.90 (0.37-2.18)	0.82	3.05 (0.83-11.13)	0.09

## Discussion

In our study, compared to the control group, non-fatal MI was significantly higher in the RA group. The RR for fatal MI in RA is 3.05 compared to the control group. The incidence of MI was comparable between MI and DM. Results from previous studies have highlighted that all-cause mortality rates or specifically due to CV events are higher among patients with RA or DM than the general population [9,14]. A nationwide Danish study reported that the risk of MI for DM and RA patients was comparable [15]. Similarly, a large study from the United States found that the risks of CVD were increased in both groups, although a greater risk was observed in patients with RA as compared to those with DM [16]. An increased risk of sudden death, silent ischemic heart disease, and more often death shortly after developing heart failure was observed in RA patients compared to the control group [17]. A Taiwanese found that RA patients more often experienced adverse outcomes than the control group [18].

Several mechanisms including individual differences in pain perception and generalized hyposensitivity to myocardial ischemia play an important role in the pathology of RA, and more recently, the balance between proinflammatory and anti-inflammatory cytokines [19-21]. According to the inflammation-based hypothesis, there is an increased production of anti-inflammatory cytokines with decreasing expression of CD11b/CD18 adhesion molecules on phagocytes among patients with asymptomatic ischemia [20]. Angina may be equally experienced in the RA and the non-RA subjects, but the RA patients usually neglect this symptom or may be less likely to consult a physician for this symptom, considering it is associated with arthritis. Their physicians may not pay attention to this and do not consider the problem as being of cardiac origin. Further investigation is required because the long-term prognosis after an unrecognized MI may result in worse outcomes as compared to the recognized MI [19,20].

To the best of our knowledge, this is the first study in the regional setting to compare diabetic and RA patients for incidence of MI. However, a further large-scale study is needed to assess the impact of RA disease on risk factors associated with MI. Moreover, due to infarctions in RA patients, they usually present with minimal or no symptoms, which ultimately leads to sudden death as observed in our study. Due to their pathologic mechanism, which results in myocardial ischemia, the management of RA patients should be multidisciplinary, including cardiologists. In addition to this, patients who are at risk of CVD should be monitored regularly to avoid the worst prognosis.

## Conclusions

In this study, we found that the incidence of MI is comparable in patients with RA and diabetes and is more in RA patients compared to the general population. Comparison between RA and DM, both of which are directly associated with increased mortality rates, is useful when evaluating the importance of risk factors influencing overall health and longevity and planning appropriate interventions. The management of these patients should be multidisciplinary, including cardiologists. In addition to this, patients who are at risk of CVD should be monitored regularly to avoid the worst prognosis.

## References

[1] Silman AJ, Pearson JE (2002). Epidemiology and genetics of rheumatoid arthritis. Arthritis Res Ther.

[2] Guo Q, Wang Y, Xu D, Nossent J, Pavlos NJ, Xu J (2018). Rheumatoid arthritis: pathological mechanisms and modern pharmacologic therapies. Bone Res.

[3] Cojocaru M, Cojocaru IM, Silosi I, Vrabie CD, Tanasescu R (2010). Extra-articular manifestations in rheumatoid arthritis. Maedica (Bucur).

[4] Maradit-Kremers H, Crowson CS, Nicola PJ, Ballman KV, Roger VL, Jacobsen SJ, Gabriel SE (2005). Increased unrecognized coronary heart disease and sudden deaths in rheumatoid arthritis: a population-based cohort study. Arthritis Rheum.

[5] Van Doornum S, McColl G, Wicks IP (2002). Accelerated atherosclerosis: an extraarticular feature of rheumatoid arthritis?. Arthritis Rheum.

[6] Sattar N, McCarey DW, Capell H, McInnes IB (2003). Explaining how "high-grade" systemic inflammation accelerates vascular risk in rheumatoid arthritis. Circulation.

[7] Poulsen MK, Henriksen JE, Dahl J (2010). Left ventricular diastolic function in type 2 diabetes mellitus: prevalence and association with myocardial and vascular disease. Circ Cardiovasc Imaging.

[8] Chiha M, Njeim M, Chedrawy EG (2012). Diabetes and coronary heart disease: a risk factor for the global epidemic. Int J Hypertens.

[9] Rao Kondapally Seshasai S, Kaptoge S, Thompson A (2011). Diabetes mellitus, fasting glucose, and risk of cause-specific death. N Engl J Med.

[10] Tancredi M, Rosengren A, Svensson A-M (2015). Excess mortality among persons with type 2 diabetes. N Engl J Med.

[11] Shah AD, Langenberg C, Rapsomaniki E (2015). Type 2 diabetes and incidence of cardiovascular diseases: a cohort study in 1·9 million people. Lancet Diabetes Endocrinol.

[12] Houge IS, Hoff M, Thomas R, Videm V (2020). Mortality is increased in patients with rheumatoid arthritis or diabetes compared to the general population - the Nord-Trøndelag Health Study. Sci Rep.

[13] Magda S (2010). Rheumatoid arthritis vs. diabetes mellitus as risk factors for cardiovascular disease: the CARRE study. Maedica (Bucur).

[14] Meune C, Touzé E, Trinquart L, Allanore Y (2009). Trends in cardiovascular mortality in patients with rheumatoid arthritis over 50 years: a systematic review and meta-analysis of cohort studies. Rheumatology (Oxford).

[15] Lindhardsen J, Ahlehoff O, Gislason GH, Madsen OR, Olesen JB, Torp-Pedersen C, Hansen PR (2011). The risk of myocardial infarction in rheumatoid arthritis and diabetes mellitus: a Danish nationwide cohort study. Ann Rheum Dis.

[16] Curtis JR, Yang S, Singh JA (2018). Is rheumatoid arthritis a cardiovascular risk-equivalent to diabetes mellitus?. Arthritis Care Res (Hoboken).

[17] Gabriel SE (2008). Cardiovascular morbidity and mortality in rheumatoid arthritis. Am J Med.

[18] Lai C-H, Hsieh C-Y, Barnado A (2020). Outcomes of acute cardiovascular events in rheumatoid arthritis and systemic lupus erythematosus: a population-based study. Rheumatology (Oxford).

[19] Cohn PF, Fox KM, Daly C (2003). Silent myocardial ischemia. Circulation.

[20] Mazzone A, Cusa C, Mazzucchelli I (2001). Increased production of inflammatory cytokines in patients with silent myocardial ischemia. J Am Coll Cardiol.

[21] Li J-J (2004). Silent myocardial ischemia may be related to inflammatory response. Med Hypotheses.

